# Provider‐level variation in the delivery of primary care telehealth for the rural Medicare Advantage population

**DOI:** 10.1111/jrh.70127

**Published:** 2026-02-04

**Authors:** Debra Bozzi, Amanda Sutherland, Melanie Canterberry, Emily Boudreau, Gosia Sylwestrzak

**Affiliations:** ^1^ Humana Healthcare Research LLC Louisville Kentucky USA

**Keywords:** Medicare Advantage, primary care, rural, telehealth

## Abstract

**Purpose:**

The role of telehealth use in primary care among rural Medicare Advantage (MA) beneficiaries following Medicare's expanded telehealth coverage during COVID‐19 is not well understood. With increasing evidence that provider characteristics influence patient access to telehealth, this study compared receipt of telehealth primary care between rural and nonrural MA beneficiaries by providers’ level of telehealth delivery.

**Methods:**

Using claims for MA beneficiaries from January 2021 to June 2024, we compared the proportion of primary care visits that were delivered via telehealth between rural and nonrural beneficiaries. We then categorized primary care physician (PCP) groups into quartiles based on the provision of telehealth as a share of total primary care visits. We conducted visit‐level generalized linear regression analyses to assess whether differences in telehealth primary care receipt between rural and nonrural beneficiaries varied by PCP telehealth quartile.

**Findings:**

PCPs delivering the highest rates of telehealth were significantly more likely to provide primary care via telehealth to rural MA beneficiaries than nonrural ones (4th quartile odds ratio: 1.12, 95% confidence interval: 1.02, 1.22). This finding differed from the overall disparity in telehealth use between rural and nonrural populations, in which rural beneficiaries used less telehealth.

**Conclusions:**

Results showing increased telehealth use among rural MA beneficiaries receiving care from PCPs delivering the highest rates of telehealth may partly stem from unique capabilities among these providers, who are potentially better equipped with tools for implementing telehealth. As such, we provide insight on provider‐oriented factors that can bolster telehealth access for rural MA populations.

## INTRODUCTION

Rural Medicare beneficiaries often face challenges accessing primary care, with contributing factors including workforce shortages among advanced primary care clinicians and high transportation costs for patients traveling long distances to receive care.^1^, ^2^, ^3^ Telehealth addresses some of these challenges by providing a platform for patients to receive medical care without the need for transportation.^2^ Medicare's expanded telehealth coverage during COVID‐19 accelerated telehealth adoption as a mechanism for delivering primary care,^4^, ^5^ but disparities in its utilization persist, with rural patients continuing to utilize telehealth for primary care at lower rates.^6^, ^7^, ^8^, ^9^

Recent studies have demonstrated a rise in the use of telehealth in primary care among the overall Medicare population since the onset of the COVID‐19 pandemic and adoption of expanded pandemic‐era telehealth flexibilities, but uptake has varied widely by region and enrollee characteristics.^10^, ^11^ Certain groups with potential access challenges—such as low‐income status, disability, and frailty—were more likely to use telehealth for primary care visits compared to individuals without these characteristics;^12^ notably, however, this pattern was not observed among beneficiaries living in rural areas, who remained less likely to use telehealth for primary care services.^6^, ^7^, ^8^, ^9^ Research regarding limited use of telehealth for primary care visits among rural enrollees suggests insufficient resources and access to care in rural areas as contributors, rather than patients’ unwillingness to use these services.^7^ In fact, survey data has shown that nearly three‐quarters of rural patients have favorable perceptions of telehealth visits, but technology issues and gaps in care coordination serve as barriers to adoption.^13^ For rural beneficiaries enrolled in Medicare Advantage (MA), the narrative may be even more complex, with one study finding that despite greater access to telehealth services among MA beneficiaries, these individuals were less likely to use telehealth services than their traditional Medicare counterparts.^14^ As some pandemic‐era telehealth flexibilities are set to expire on January 30, 2026,^15^ there remains uncertainty about how these policy shifts will affect the availability and use of telehealth among rural and nonrural MA enrollees.

Emerging work demonstrates the increasingly crucial role of primary care physicians (PCPs) in the adoption of telehealth services for their patients, but existing research has not yet explored the provider‐driven impact on access to telehealth in the primary care setting within the rural MA population.^12^, ^16^, ^17^ With increasing evidence that provider characteristics influence patient access to telehealth, our study builds on prior investigations by comparing receipt of telehealth primary care between rural and nonrural MA beneficiaries by providers’ relative level of telehealth delivery.

## METHODS

We used claims for MA beneficiaries from Humana Inc. enrolled in Health Maintenance Organization (HMO) and Preferred Provider Organization (PPO) plans from January 2021 to June 2024. Eligible beneficiaries were included for each year of the study period in which they were enrolled in a plan. Beneficiaries were excluded from the study if their plan was contractually excluded from research or their assigned primary care provider delegated claims to a third party, since the MA plan administrator does not have full access to delegated claims. Beneficiaries were excluded if they were in institutionalized care during the study period. Rural status was defined using a Rural Urban Commuting Area (RUCA) code of 4 or greater based on residential zip code. Additional detail on the cohort is available in the Supporting Information.

We identified in‐person and audio‐visual telehealth outpatient encounters with a PCP using place of service (POS), procedure code, and provider specialty. We classified visits as audio‐visual telehealth using POS codes 02 or 10 and procedure modifier codes of 95, GT, or GQ. PCP groups were identified by taxpayer identification number and categorized into quartiles based on provision of telehealth as a share of total primary care visits in each calendar year, such that telehealth was more common among PCP groups in the highest quartile. Providers with at least 100 encounters in a given calendar year and telehealth use greater than 0% and less than 100% were included. Additional detail on the identification of in‐person and audio‐visual primary care outpatient encounters and assignment to PCP quartile can be found in the Supporting Information.

We descriptively compared the proportion of primary care visits that were telehealth between rural and nonrural beneficiaries, within each PCP quartile. Next, we performed visit‐level generalized linear regression analyses with a binomial distribution and logit link function to assess whether differences in telehealth receipt between rural and nonrural beneficiaries varied by PCP telehealth provision quartile. The outcome for each model was whether the primary care visit was delivered via telehealth or not. The independent variable of interest was an interaction between rural status and PCP quartile. Beneficiary‐level covariates included age, sex, race, low‐income status, and disability as the original reason for Medicare entitlement. Race was assessed according to the Centers for Medicare and Medicaid Services (CMS) beneficiary race code and categorized as Black, White, underrepresented (Asian, Hispanic, North American Native, and Other), and unknown. Models also included month fixed effects, and standard errors were clustered by state.

Additional analyses are available in the Supporting Information. We compared monthly trends in the share of primary care visits that were telehealth between rural and nonrural beneficiaries, by PCP quartile, to illustrate whether differences in telehealth utilization varied over time. We also examined the distribution of primary care telehealth visits by POS code (at‐home vs. not) for rural and nonrural beneficiaries by PCP quartile in 2024, to better understand how beneficiaries used these services.

## RESULTS

Among 5,475,860 MA beneficiaries, 25.42% lived in a rural zip code, while the remaining 74.58% lived in a nonrural zip code. Rural beneficiaries experienced a lower rate of telehealth as a share of primary care visits compared to nonrural beneficiaries (4.41% vs. 5.86%; Table 1).

**TABLE 1:** Beneficiary characteristics.

	Full cohort	Rural	Nonrural	SMD
Sample size, *N* (%)	5,475,860 (100.00)	1,392,037 (25.42)	4,083,823 (74.58)	
Age; mean (SD)	70.90 (10.20)	70.20 (10.40)	71.10 (10.20)	−0.09
Female, *n* (%)	3,088,888 (56.41)	767,895 (55.16)	2,320,993 (56.83)	−0.03
Race, *n* (%)				
Black	1,073,647 (19.61)	204,404 (14.68)	869,243 (21.29)	−0.17
White	3,967,550 (72.46)	1,117,722 (80.29)	2,849,828 (69.78)	0.24
Underrepresented	298,337 (5.45)	43,664 (3.14)	254,673 (6.24)	−0.15
Unknown	136,326 (2.48)	26,247 (1.89)	110,079 (2.69)	−0.05
Low‐income status, *n* (%)	1,843,73 (33.67)	540,043 (38.80)	1,303,710 (31.92)	0.14
Disability, *n* (%)	1,899,558 (34.69)	567,512 (40.77)	1,332,046 (32.62)	0.17
Plan type, *n* (%)				
HMO	2,485,379 (45.39)	401,426 (28.84)	2,083,953 (51.03)	−0.44
PPO	2,990,481 (54.61)	990,611 (71.16)	1,999,870 (48.97)	0.44
Share of primary care visits delivered via telehealth (%)	5.50	4.41	5.86	−0.7

Abbreviations: HMO, health management organization; PPO, preferred provider organization.

**FIGURE 1:**
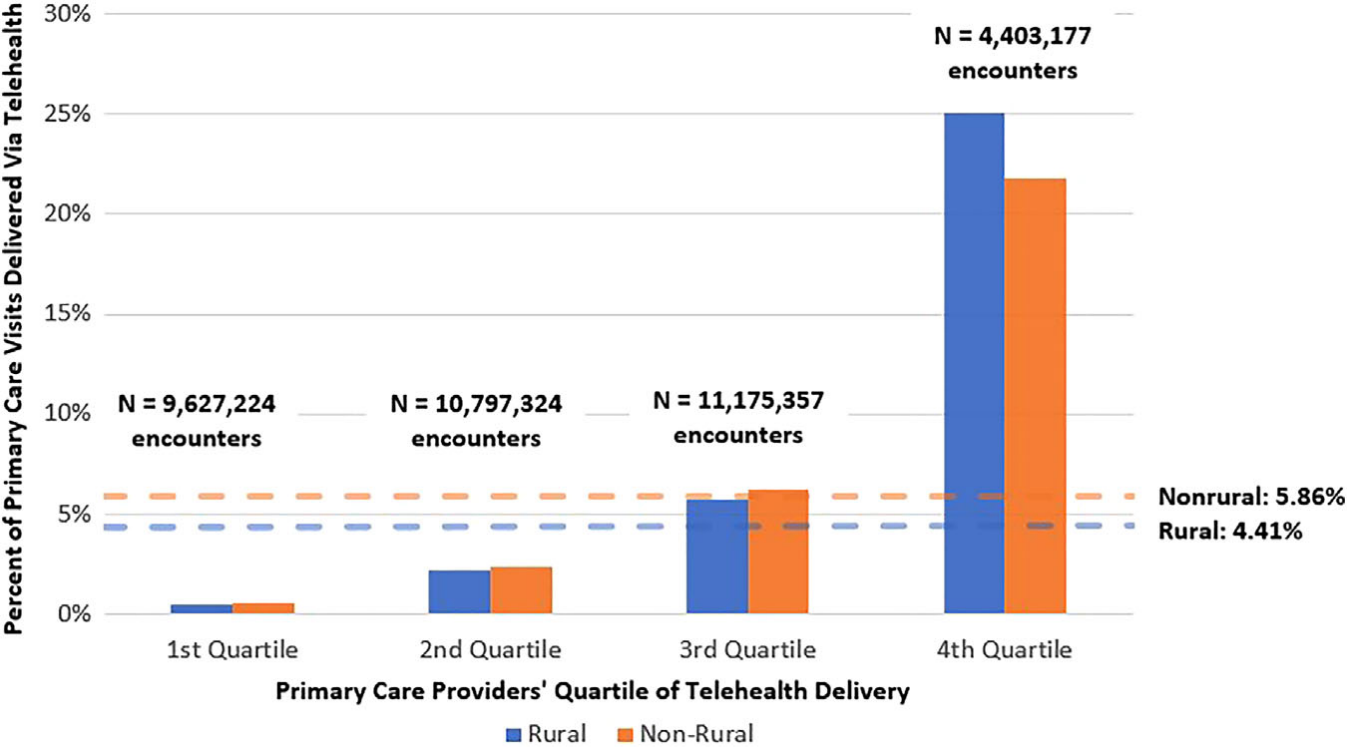
Unadjusted rate of telehealth primary care receipt as a share of primary care visits among rural vs. nonrural beneficiaries, by PCP quartile of telehealth delivery.

Descriptively, rural beneficiaries receiving care from PCPs in the bottom three quartiles of telehealth provision also utilized lower rates of telehealth than their nonrural counterparts. Conversely, rural beneficiaries receiving care from PCPs delivering the highest level of telehealth (4th quartile) used a higher proportion of telehealth relative to nonrural beneficiaries (25.00% vs. 21.80%; Figure 1). PCP groups in the 4th quartile had the fewest number of encounters, representing 12.23% of the total number of encounters.

**FIGURE 2:**
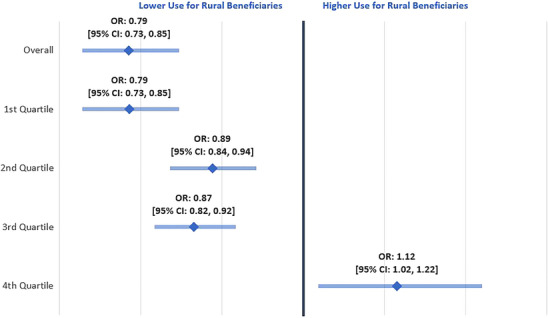
Odds of telehealth primary care receipt among rural (compared to nonrural) beneficiaries, by PCP quartile of telehealth delivery.

In adjusted models, rural beneficiaries overall had lower odds of telehealth use versus nonrural beneficiaries (Odds Ratio (OR): 0.79, 95% confidence interval (CI): 0.73, 0.85). Rural beneficiaries also had decreased odds of telehealth use versus nonrural beneficiaries when receiving care from PCPs delivering low telehealth rates (1st quartile OR: 0.79, 95% CI: 0.73, 0.85; 2nd quartile OR: 0.89, 95% CI: 0.84, 0.94; 3rd quartile OR: 0.87, 95% CI: 0.82, 0.92), but significantly higher odds of telehealth receipt when obtaining care from PCPs with high telehealth rates (4th quartile OR: 1.12, 95% CI: 1.02, 1.22; Figure 2).

## DISCUSSION

In this study of nearly 5.5 million MA beneficiaries, we compared the receipt of telehealth primary care between rural and nonrural beneficiaries by providers’ relative level of telehealth delivery. We found that PCPs delivering the highest rates of telehealth were more likely to provide primary care via telehealth to rural MA beneficiaries than nonrural ones. This finding differed from the overall disparity in telehealth use between rural and nonrural populations, in which rural beneficiaries used less telehealth.

This result may partly stem from unique capabilities among providers delivering high rates of telehealth, who might be better equipped with tools for implementing telehealth and appropriate training to understand experiences of rural beneficiaries. Our findings are consistent with recent work showing that PCPs with increased telehealth‐delivered outpatient visits see more medically and socially complex patients in traditional Medicare.^17^ The present study furthers and strengthens those findings, which did not evaluate MA or rural populations by PCPs’ degree of telehealth provision.

Our findings align with external literature illustrating that overall, rural individuals use less telehealth primary care than nonrural ones.^6^, ^7^, ^8^, ^9^ Earlier studies are mixed regarding the mechanism for lower use of telehealth primary care among rural enrollees. Select studies cited insufficient resources and access as contributors, rather than patients’ unwillingness to use these services,^7^ while others have observed reduced telehealth use among enrollees even with greater availability of these services.^14^ Notably, our study is among the first to provide insight on provider‐oriented factors that bolster telehealth access for the rural MA population. Our findings suggest that MA insurers and policymakers have potential to address primary care access challenges for rural MA beneficiaries—a historically intractable issue—by ensuring that in‐network PCPs are empowered to deliver these services via telehealth.^18^

This study has important implications as several pandemic‐era telehealth flexibilities—which allowed Medicare beneficiaries to access telehealth services from any setting, including their home—are set to expire in 2026. These changes largely represent a reversion to pre‐pandemic telehealth policy, with coverage contingent on patient location. Moving forward, most telehealth visits will require patients to be in rural areas or federal telehealth demonstration sites, and home‐based visits will no longer qualify (with exceptions for behavioral health services). Instead, patients will need to be located in an eligible setting (e.g., a physician's office, hospital, or other health care facility).^15^ The rationale for this shift is 2‐fold: financial sustainability^19^, ^20^ and concerns that home‐based visits limit collection of vitals and other clinical data, reducing the value of the visit.^21^, ^22^ Although the impact of this change remains uncertain—and adoption of these policies may vary across MA plans—it will likely reduce overall utilization of telehealth primary care. Our data (see Supporting Information) demonstrated that rural beneficiaries had a slightly lower proportion of primary care telehealth visits billed with an at‐home POS code in 2024, across all levels of providers’ telehealth delivery, suggesting a smaller impact on this population. Future studies exploring differential impacts on rural and nonrural MA beneficiaries’ telehealth use, as well as their providers, would offer clearer insight on how telehealth will be utilized amid an evolving policy landscape.

This analysis has several limitations. First, this study was limited to MA beneficiaries from one insurer, which may not reflect telehealth utilization among the broader Medicare population. Although the health plan serves nearly one‐fifth of the MA population, results may not be representative of other demographic cohorts, including patients enrolled in traditional Medicare. Generalizability may also vary by provider groups’ level of telehealth provision. For example, provider groups with the highest level of telehealth delivery had the fewest number of encounters, which is possibly due to relatively smaller Humana membership than provider groups with lower levels of telehealth delivery. Second, data included few provider characteristics, limiting potential subpopulation analyses. For example, we were unable to determine the provider's precise location, which could provide additional insight on how telehealth is delivered to rural versus nonrural beneficiaries. Third, we did not adjust for clinical risk in the adjusted models, as by construction, this measure would require an extended continuous enrollment period, thereby reducing the study population. Fourth, this study did not focus on changes over time in primary care telehealth use between rural and nonrural beneficiaries by PCP groups’ provision of telehealth. We present monthly trends in primary care telehealth utilization by PCP quartile in the Supporting Information, largely show that the share of telehealth primary care visits tracked together throughout the entire study period. Thus, our main finding emphasizes differences in levels, rather than temporal shifts, between these groups. Future research should revisit these trends following the expiration of several Medicare telehealth flexibilities on January 30, 2026. Finally, this study was observational, so we could not determine causality. Future research should explore mechanisms enabling certain providers to deliver greater levels of telehealth to rural enrollees.

Given the barriers to receiving primary care among rural patients and the potential for telehealth to improve access, it is important to understand factors contributing to the lower use of telehealth typically observed among this population. We found that the share of primary care delivered via telehealth is higher among rural compared to nonrural MA beneficiaries when the PCP group has high telehealth uptake, suggesting that the lower telehealth rates typically observed may be partly driven by access at the provider level.

## CONFLICT OF INTEREST STATEMENT

The authors report no conflicts of interest.

## Supporting information



Supporting Information
