# Supplementary material for: Provider‐level variation in the delivery of primary care telehealth for the rural Medicare Advantage population
Source: J Rural Health. 2026 Feb 4;42(1):e70127. doi: 10.1111/jrh.70127 (PMC12870281; doi:10.1111/jrh.70127)
Supplement: Supplementary file 1 — Supporting Information [file JRH-42-0-s001.docx]

**Supporting Information: Provider-Level Variation in the Delivery of Primary Care Telehealth for the Rural Medicare Advantage Population**

Definitions

*Sample:*

Humana Medicare Advantage beneficiaries enrolled in Health Maintenance Organization (HMO) and Preferred Provider Organization (PPO) plans were included. Eligible beneficiaries were included for each year of the study period they were enrolled in a plan.

Beneficiaries were excluded from the study if their plan was contractually excluded from research or their assigned primary care provider delegated claims to a third party, since the MA plan administrator does not have full access to delegated claims. Beneficiaries were excluded if they were in institutionalized care during the study period.

*Primary Care Visit Identification*

This analysis used outpatient claims for a clinician visit with a primary care provider that were identified as either in-person or telehealth.

1. Outpatient claims were identified by POS codes (listed below):

- 02, 05, 06, 10, 11, 12, 13, 14, 20, 22, 24, 32, 33, 34, 49, 50, 71

1. Clinician visits were identified by CPT/HCPCS codes (listed below).

- G0101, G0245, G0248, G0402, 0500F, 99201, 99202, 99203, 99204, 99205, 99381, 99382, 99383, 99384, 99385, 99386, 99387, G0246, G0247, G0250, G0420, G0421, G0463, 0502F, 0504F, 2000F, 95115, 95117, 99058, 99211, 99212, 99213, 99214, 99215, 99354, 99355, 99366, 99367, 99391, 99392, 99393, 99394, 99395, 99396, 99397, 99401, 99402, 99403, 99404, 99411, 99412, 99420, 99429

1. Clinician visits delivered by a primary care practitioner were determined by the billing NPI’s HIPPA provider taxonomy (included classification and specializations listed below).
2. Audio-visual telehealth visits were determined by POS or CPT modifier as a subset of primary care visits (listed below).

- POS: 2, 10
- CPT Modifier: 95, GT, GQ
- Note: Audio-only visits (CPT codes 99441-99443) were not included.

| **Primary Care** | |
| --- | --- |
| **HIPPA Taxonomy Class/Specialization Combinations** | |
| **Class** | **Specialization** |
| **General Practice** |  |
| **Nurse Practitioner** |  |
| **Physician Assistant** |  |
| **Family Medicine** |  |
| **Internal Medicine** | **No Specialization (must be blank)** |
| **Internal Medicine** | **Geriatric Medicine** |
|  | **Community Health** |
|  | **Primary Care** |
|  | **Public Health, Federal** |
|  | **Public Health, State of Local** |

*Primary Care Provider Group Quartile*

For each encounter, we identified the primary care provider group (Tax ID level) at the visit level. Then, we calculated the proportion of primary care visits delivered via telehealth for each PCP group, in each calendar year. We excluded PCP groups with fewer than 100 encounters in a given calendar year. We also excluded PCP groups with no telehealth visits (0% telehealth) and those with only telehealth visits (100% telehealth) as a share of primary care visits in a calendar year. We then categorized PCP groups into quartiles based on the share of primary care visits delivered via telehealth. Quartiles were calculated separately in each calendar year. Provider quartile upper bounds are shown in the table below:

| **Provider quartile upper bounds** | | | | |
| --- | --- | --- | --- | --- |
|  | **2021** | **2022** | **2023** | **2024** |
| Quartile 1 | 1.54% | 1.31% | 0.90% | 0.88% |
| Quartile 2 | 4.79% | 4.05% | 2.93% | 2.70% |
| Quartile 3 | 12.55% | 10.34% | 8.22% | 7.35% |
| Quartile 4 | 99.75% | 99.86% | 99.75% | 99.33% |

**Regression Equations**

***Variables:***

- **Telehealth Use** $\boldsymbol{(}\boldsymbol{Y}_{\boldsymbol{it}}\boldsymbol{)}$ **=** Binary outcome variable where 1 represents a telehealth visit and 0 represents a non-telehealth visit
- **Disability =** Binary indicator where 1 represents disability as the original reason for Medicare entitlement
- **Age =** Continuous variable for beneficiary age at the start of a given calendar year
- **Gender =** Categorical variable for beneficiary gender
- **Race =** Categorical variable for beneficiary race
- **Rural =** Binary indicator where 1 represents beneficiaries living in rural areas in a given year
- **Low-Income Status (LIS/DE)=** Binary indicatory where 1 represents beneficiaries eligible to receive a low-income subsidy and/or dually eligible for Medicare and Medicaid in a given year
- **State:** Categorical variable for beneficiary state
- **PCP Quartile =** Categorical variable for a PCP group’s assigned quartile in a given calendar year, based on the share of primary care visits delivered via telehealth.

**Indices:**

- i = Beneficiary-level
- t = Year-level

**Notes (all models)**

- standards errors clustered by State
- time fixed effects (year)
- Link: logit
- Distribution: binomial

| **Rural x PCP Quartile** | $\boldsymbol{Y}_{\boldsymbol{it}}\boldsymbol{=}\boldsymbol{B}_{\boldsymbol{0}}\boldsymbol{+}\boldsymbol{B}_{\boldsymbol{1}}\boldsymbol{\cdot}\boldsymbol{Disability}_{\boldsymbol{i}}\boldsymbol{+}\boldsymbol{B}_{\boldsymbol{2}}\boldsymbol{\cdot}\boldsymbol{Age}_{\boldsymbol{it}}\boldsymbol{+}\boldsymbol{B}_{\boldsymbol{3}}\boldsymbol{\cdot}\boldsymbol{Gender}_{\boldsymbol{i}}\boldsymbol{+}\boldsymbol{B}_{\boldsymbol{4}}\boldsymbol{\cdot}\boldsymbol{Race}_{\boldsymbol{i}}\boldsymbol{+}\boldsymbol{B}_{\boldsymbol{5}}\boldsymbol{\cdot}\boldsymbol{Rural*PCP Quartile}_{\boldsymbol{it}}\boldsymbol{+}\boldsymbol{B}_{\boldsymbol{6}}\boldsymbol{\cdot}\boldsymbol{LIS/DE}_{\boldsymbol{it}}\boldsymbol{+}\boldsymbol{B}_{\boldsymbol{7}}\boldsymbol{\cdot}\boldsymbol{Year}_{\boldsymbol{t}}\boldsymbol{+}\boldsymbol{B}_{\boldsymbol{8}}\boldsymbol{\cdot}\boldsymbol{State}_{\boldsymbol{it}}$ |
| --- | --- |

**Monthly Trends in the Share of Primary Care Telehealth Visits Among Rural vs. Nonrural Beneficiaries, By PCP Quartile**

**
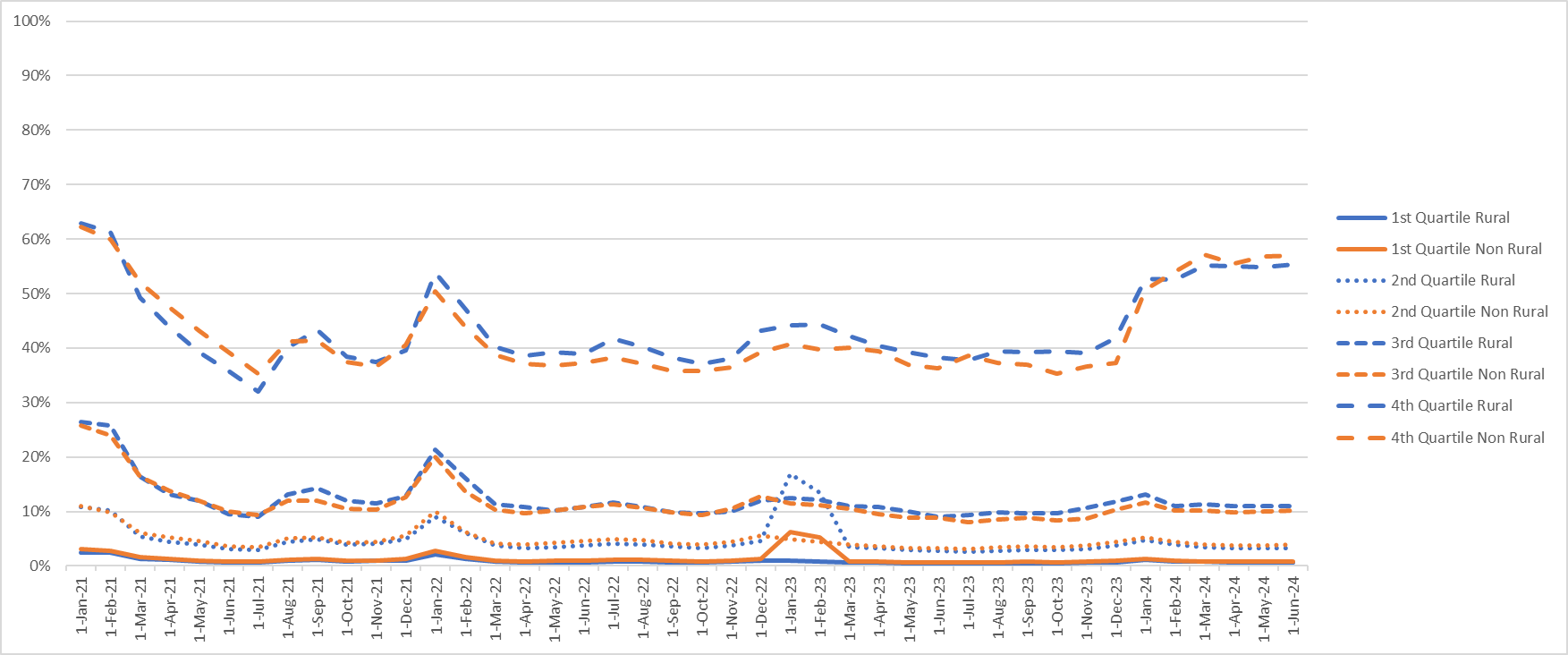
**

**Share of Telehealth Primary Care Visits With At-Home POS vs. Non-Home POS, 2024**

**
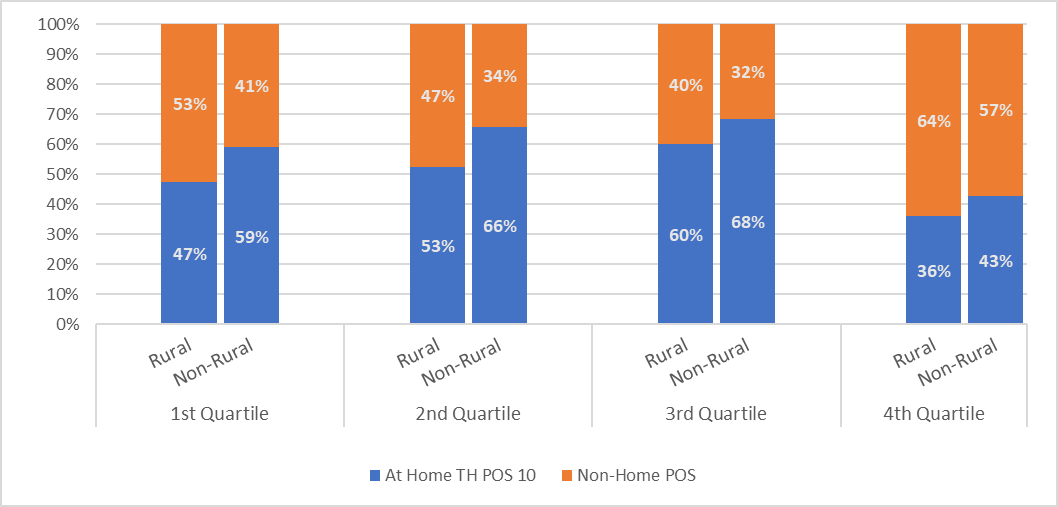
**

At Home POS was defined as POS = 10; Non-Home POS was defined by all other POS codes.
